# Meals on wheels services and the food security of older people

**DOI:** 10.1111/hsc.14092

**Published:** 2022-10-27

**Authors:** Angela Dickinson, Wendy Wills

**Affiliations:** ^1^ Centre for Research in Public Health and Community Care University of Hertfordshire Hatfield UK

**Keywords:** community meals, covid‐19, food practices, food security, meals on wheels, older people, relationship centred care

## Abstract

In recent years, Meals on Wheels (MoW) services have been in a state of decline as austerity policies have become entrenched. However, this decline is occurring with little knowledge of the impact withdrawal of MoW services has on the health and well‐being of those who use them. The pandemic has raised awareness of precarity and vulnerability in relation to food that affects many people in the UK and other Westernised countries and this provides further context for the analysis presented. This paper presents findings of a mixed methods ethnographic study drawing on qualitative interviews and visual methods underpinned by social practice theory to explore the household food practices of older people receiving MoW services. Interviews were conducted with 14 older people receiving MoW, eight MoW staff delivering MoW services in the east of England and one expert. The Covid‐19 pandemic interrupted the study, and once the first lockdown began visits to the homes of older people were terminated and the remaining interviews were undertaken by telephone. The study found that a number of threats accumulated to change food practices and moved people towards vulnerability to food insecurity. Threats included difficulty accessing food and cooking due to sensory and physical challenges. The MoW service increased participants' coping capacity. As well as benefiting from the food provided, the relational aspect of the service was important. Brief encounters between MoW staff built caring relationships that developed over time to ensure older people felt valued and cared for. The study demonstrates how MoW services make a positive contribution to food practices, supporting vulnerable adults to continue living well in their own homes and protecting them from food insecurity and ill‐being. Local authorities looking to make cost savings through ending MoW services should consider the impact this would have on the well‐being of older residents.

**What is known about this topic and what this paper adds?**

Please provide up to three bullet points on what is known about this topic, and three bullet points on what the paper adds.

**What we know?**

- Older people are at risk of experiencing food insecurity and malnutrition.
- MoW services are in decline in the UK as a result of austerity policies.
- We know very little about experiences of receiving MoW services in the UK and how they affect older people's food practices and food security.

**What the paper adds?**

- Provides an analysis of how ageing can impact on household food practices and make households vulnerable to food insecurity
- Adds understanding of how an effective MoW service can support social well‐being and strengthen the coping capacity
- The paper explores how MoW can shift older people away from food insecurity and support older people to live independently in their own homes.

## 1 INTRODUCTION

Worldwide demographic shifts and increasing longevity mean many countries have an increasing number of older people, which, while being a cause for celebration, creates challenges for health and social care systems. Ageing is associated with high levels of heterogeneity in the population, but older people are disproportionally affected by food insecurity and malnutrition (Dickinson et al., 2021). Malnutrition is associated with factors such as poor mobility (Coveney & O'Dwyer, 2009), chemosensory losses (Boyce & Shone, 2006; Dickinson et al., 2014), difficulties with swallowing and issues such as loneliness (Burris et al., 2021) and changes to mental health, for example, cognitive impairment (Volkert et al., 2019). In the UK, pre‐pandemic, around one in 10 people aged over 65 were affected by malnutrition (Malnutrition Task Force, 2017). The estimated annual cost to the health economy of malnutrition in 2017 was £23.5 billion for older people and malnutrition in older people contributed half of this cost (Stratton et al., 2018). These figures are likely to have increased during the pandemic as older people have been disproportionately affected by the virus, as those aged over 70 and with particular medical conditions were advised to ‘shield’ or stay at home as much as possible to reduce exposure to the Covid‐19 virus (UK Cabinet Office, 2020). This mitigation has had adverse consequences on other aspects of well‐being (Sustain, 2020).

Historically, one intervention aiming to provide hot ready prepared food to those who are no longer able to cook meals for themselves has been the provision of meals on wheels (MoW) services. The MoW service was initially developed in the UK in Hertfordshire following World War II by the Women's Royal Voluntary Service. The ‘wheels’ in the name referred to the prams originally used to transport hot meals to those who needed them (Campbell et al., 2015). Meals on Wheels services expanded across the UK (and then to other countries including the US, Canada, Australia and New Zealand), providing food deliveries to the homes of people who would otherwise not be able to access a hot meal, with many local authorities delivering the service to their residents in return for a small fee paid by the recipient. MoW services have been shown to increase nutrient intake and offer a number of other benefits including providing opportunities for welfare checks, reducing loneliness and isolation through providing opportunities for social interaction and increasing quality of life (Thomas et al., 2015; Zhu & An, 2013). A recent evaluation found that MoW were able to reverse malnutrition experienced by older people (Dewar et al., 2020). Evidence from the US shows that MoW have a positive impact, playing an important role in preventative care evidenced by preventing admission to residential care by supporting people to live independently in their own homes (Thomas & Mor, 2013). In addition, they can help alleviate depression and support the maintenance of a healthy weight (Kim & Frongillo, 2007). Cuts in funding for public health have reduced the availability of services that focus on supportive and preventative services often with deprived areas being worst hit (Oliver, 2022). This has impacted on MoW services which have been in decline in the UK as local authority budgets have been cut as a result of austerity policies, with no statutory protection for such interventions. This has resulted in the patchy provision and the most recently available data shows that only 42% of local authorities provided MoW services (National Association of Care Catering, 2018), a fall from 66% in 2014. Recent work exploring MoW provision in London found that MoW services were inadequate before the pandemic (Sustain, 2020). A recent study found that the pandemic was placing an increasing demand on MoW services (Papadaki et al., 2021). However, further research evidence is needed to understand the value or cost‐effectiveness of the service and how these impact on the food security of older people (Sustain, 2020).

Food insecurity is defined as ‘the inability to consume an adequate quality or sufficient quantity of food in socially acceptable ways, or the uncertainty that one will be able to do so’ (Dowler & O'Connor, 2012, p45). Meals on wheels have a clear role to play in maintaining the food security of older people in an acceptable way for those unable to shop or cook for themselves (Warren et al., 2020). Recently there has been increasing concern about food insecurity in later life. These include the establishment of an All Party Parliamentary Group on hunger and malnutrition in England (All‐Party Parliamentary Group on Hunger, 2018), and the Malnutrition and Prevention Network (MAPN) which brings together academics, health, social care and voluntary sector professionals and others working to prevent and alleviate malnutrition (https://www.malnutritiontaskforce.org.uk/malnutrition‐awareness‐prevention‐network) and the Meals on Wheels Alliance (hosted by Sustain).

In this paper, we report on a study underpinned by a practice theory approach (Wills et al., 2015). A practice approach provides a way to re‐orientate away from public health approaches that focus on concepts of behaviour which assume rational choices made by individuals (Cohen & Cribbs, 2017). Food practices are performed every day and are such a routine and embedded aspect of daily life that they become tacit, having been performed repeatedly and being shaped over the life course (Bourdieu, 1984; Shove et al., 2012). Food practices comprise a combination of intertwined elements associated with the actions of buying, preparing/cooking, eating, clearing away and dealing with food waste, and require the actor to draw on sets of knowledge, values, beliefs, resources, technologies and relationships (Wills et al., 2016). People are viewed as ‘carriers of practices’ (Reckwitz, 2002) and individuals are no more important than any other element that forms the practice. Importantly, the practice approach encompasses the wider set of factors that influence what people do that can make them vulnerable. Practices comprise three core components; meanings (the attributions assigned to practices by those undertaking them), materials (the resources and ‘things’ people use to perform a practice) and competencies (the knowhow and skills used) (Shove et al., 2012).

Previous work by the authors, using a practices framework (Dickinson et al., 2021) enabled a more nuanced understanding of vulnerability, revealed it to be a dynamic state and explored how it might begin, worsen or be eased. The authors developed a Food Security Framework (FSF), which provides a starting point for the study reported here. The FSF includes four domains, the first, *exposure* includes factors affecting the resources available in older age, for example, financial arrangements, education. *Threats* include ‘specific events, shocks or crises’ (Schroder‐Butterfill & Marianti, 2006), for example, bereavement or physical incapacity. *Coping capacity* incorporates the assets drawn on to support health and well‐being and is composed of three further elements; *individual capacities*, *formal support* and *social networks*, and finally, ‘*bad outcomes*’ which could range from poor health to mortality resulting from malnutrition. This paper focuses on two domains of the FSF; *threats* and *coping capacity* and aimed to understand the meaning of the MoW service to older people, and the contribution made to food security. The authors believe this is the first study to explore older people's experiences of MoW in the UK.

## 2 MATERIALS AND METHODS

The study focuses on one MoW organisation in the east of England delivering over 500,000 hot meals each year, operating as a not‐for‐profit social enterprise. Older people make a financial contribution to the service. The study involves an ethnographic (Hammersley & Atkinson, 2007) exploration of the role MoW plays within the everyday lives of older people based on observations, visual methods (photographs) and interviews (Dickinson et al., 2021; Wills et al., 2016) which generated data on everyday food practices, and how these might have changed, leading participants to use MoW services. During the visit, older people showed a researcher around their kitchen spaces. The researcher took photographs of people's kitchens, domestic appliances and household food stores. Following the kitchen tour, an informal interview was conducted using a topic guide focusing on current food practices and experiences of the MoW service.

The first lockdown of the pandemic imposed restrictions on social interactions. We adapted the methods, but tried to retain our ethnographic ethos. Interviews were carried out by telephone which meant that visual data collection was no longer possible; instead, we asked older people to describe their kitchens, and the food stored in their homes (e.g. in cupboards, fridges and freezers).

Interviews and a focus group were undertaken with staff from operational and management areas of the MoW organisation. A national MoW policy expert was invited to be interviewed to add an external perspective. Observation of an MoW round enabled a better understanding of the operation of the service.

Recruitment of older participants involved a two‐step process. The MoW provider made the first approach to a range of clients to ascertain their interest in being involved before contact details were passed to the research team. The researcher contacted participants by telephone to explain the study and if they were still willing to be involved, arranged a date and time for a home visit (latterly a telephone interview). Prior to data collection, the researcher provided written information about the study, gave time for the participant to ask questions, and gained informed consent. Participants were free to withdraw from the study at any point. Ethics approval for the study was gained from the University of Hertfordshire Health, Science, Engineering & Technology ECDA Ethics Committee (Reference: HSK/SF/UH/04706).

### 2.1 Analysis

Interviews and the focus group were transcribed and anonymised. All data sources for each individual household were read (transcripts and fieldnotes) and viewed (photographs) before being considered and discussed with the research team alongside the a priori theoretical framework in a preliminary analysis (Wills et al., 2016). All textual data were imported into NVivo™ v12 and coded to support further analysis by facilitating data management and retrieval. Data were coded deductively, and grouped according to the categories of the vulnerability framework developed from our previous work (Dickinson et al., 2021). Photographs were viewed and assisted in the theme development, for example, we were able to view Daisy's emergency food supplies in her freezer (bread, milk and other small items) alongside her descriptions of the modest, amount of food she had now had in her home to supplement the delivered meals. This enabled us to see how the amount of food stored in the home had reduced as the food was now entering the home as the food delivered by MoW service.

### 2.2 Participants

We interviewed 14 clients of the MoW service, nine were women and all were white British. Six households had a home visit (pre‐covid restrictions), and eight involved telephone interviews only. Data were collected between January and May 2020. Apart from one client who was in his 50s all participants were in their 80s and 90s and all lived alone except for one couple. We conducted one focus group with five MoW staff, interviews were undertaken with three members of staff and one MoW expert from outside the organisation. All names are pseudonyms.

## 3 RESULTS

This paper reports data on the 2 major themes of the vulnerability model, that is *threats* and *coping capacity*. All MoW participants described experiencing a number of *threats* to their food security, which led to them accessing the MoW service. They described the changes they made to their food practices as physical or mental health changed. Accessing the MoW service helped alleviate the impact of threats by strengthening their *coping capacity*, enabling them to remain independent in their own homes. Relational aspects of the MoW service were important and reduced feelings of isolation. The paper will explore these themes and, as we were collecting data as the Covid‐19 pandemic and the national lockdown began, we present analysis on how this added to worries about food insecurity, and how the MoW service helped them to feel more food secure and emotionally supported during a challenging time.

### 3.1 Threats to food security

All participants described experiencing challenges to their food security and a range of threats that affected their ability to prepare hot meals. Although most participants had the *know‐how* to prepare meals, the *skills* and/or *competence* needed to undertake a range of food‐related actions had changed. Factors that led to them struggling to access food and prepare meals ranged from increasing physical frailty, which had led to becoming housebound, functional issues following an illness such as a stroke, a bone fracture, cancer, visual impairment, cognitive issues (e.g. dementia) and a general loss of interest in food (following loss of a spouse or depression). Others realised they lacked the skills to prepare meals following an illness or the loss of a spouse who had previously undertaken household food preparation. For all participants, a number of threats had accumulated over time to shift them towards vulnerability. Examples are given below to illustrate the challenges participants experienced.

Frank tells of how his health had progressively deteriorated since his wife died 2 years ago, and how his food practices are controlled by his health conditions that impact directly on his diet. He has given up driving, uses two sticks to mobilise around his home and no longer goes out on his own:

I've got severe arthritis in both arms and hands and I find it very hard to do anything at all, even writing is very difficult. My main obstacle, my main drag on life is I've got a stoma. That more or less rules my life, what I can do, what I can eat, what I can drink, what time I go to bed, are all determined by the activity of my stoma (Frank).

Harry and Anna, a couple, had previously received frozen meal deliveries marketed to older people, but they explained that they had both become so frail neither of them was able to stand for long enough to reheat the food. Carol struggles with food shopping and carrying it home after a knee operation and Ann was assessed to be unsafe using the cooker due to visual impairments. All participants explained how they now struggled to prepare and cook ‘proper’ meals. Some still did a limited amount of food preparation, for example, preparing simple snacks and drinks, though these lacked the meaning and perceived healthier association with what participants considered to be a ‘proper’ cooked meal:

I probably wouldn't bother to cook. Well, I would cook, but probably not as I should do, if you see what I'm saying? It's so easy, isn't it? It walks through the door. I don't do anything now. I was always independent before I had this accident and broke my leg (Elsie)

Most participants described an accumulation of threats over time that had eventually meant they struggled to prepare and cook meals for themselves. Struggling to cook meals was a major threat to food security that many participants gave as the main reason given for accessing an MoW service.

### 3.2 The support offered by a meals on wheels service and the contribution to the coping capacity of older people

**FIGURE 1:**
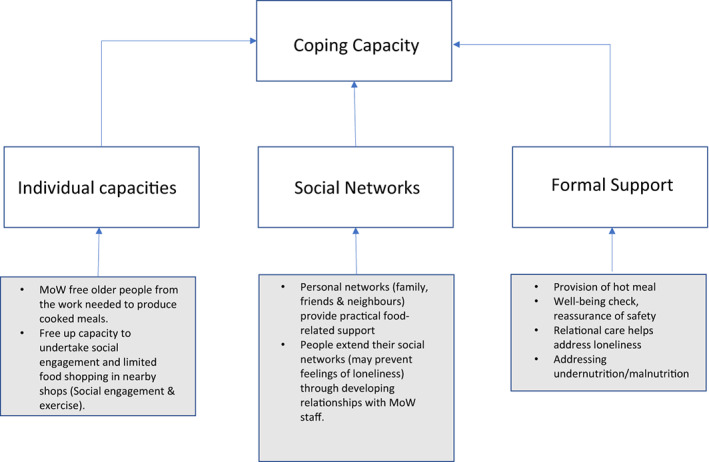
Mechanism by which MoWs support and increase the *coping capacity* of older people and strengthen resilience in relation to food security

Participants were positive and clear about the value of the MoW service as a formal support service and how this supported their coping capacity, supporting their individual capacity and enhancing their social networks (summarised in Figure 1). Participants explained that they would be unable to manage to prepare food for themselves, thus the service played a vital role in supporting them to live independently. Participants had considered or tried other forms of food provision but these were not sufficient, as they were unable to safely reheat food, including frozen food delivered to their homes or buying ready meals bought from supermarkets. Purchased ready meals were felt by participants to be of poor value nutritionally and were not conferred with the same meaning as homecooked food nor felt to be an adequate adjustment to food practices for the longer term.

Ann explains how important MoW are for her, describing the health benefit she has experienced. She started having MoW after failing a kitchen assessment during a prolonged hospital stay. She had lost a great deal of weight describing how she ‘nearly died’ and her weight dropped to five stone. She has subsequently gained weight. She believes that MoW keep her healthy, independent and living in her own home.

They're brilliant. They're important to me, and they're brilliant. I'd die if they never gave me meals. I wouldn't be able to manage. I wouldn't be able to cook for myself (Ann)

Participants saw MoW as an essential service, and they could not imagine how they would survive without it. Carol had no family nearby who could shop for her, so found the service essential.

… the older I've got and the worse the pain's got. So I don't know what I would have done if I couldn't have had Meals on Wheels. I think it's absolutely wonderful service. I was told recently that some places don't even have them. Well I wonder how on earth do the poor people manage? I mean maybe they've got family but I don't have any family here at all. My family are in [other countries]. You know, there is nobody near (Carol)

Daisy reports that MoW give her peace of mind helping her feel safe and ‘means someone is checking me every day’. She explains that the meals also ‘save my back’ as she does not have to stand in the kitchen to cook. She is very happy with the cost and says that she ‘would pay more’ for her meals. She has seen a nutritionist as part of the MoW service and thought this was beneficial. The well‐being check was seen as a vital part of the service by the following manager, particularly for those who lived alone who might not have anyone else checking on them:

There are numerous occasions where our drivers will just have that five minutes to go into somebody's house and find them on the floor and I know it sounds drastic and a bit clichéd but in that sort of situation you're potentially saving lives because nobody else has visited that elderly person that day, they might never get up off the floor. (Interview with Manager #3)

### 3.3 Meals on wheels, social networks and relational‐focused care

MoW delivery staff transport food in ‘hot boxes’ that have to be delivered over a 2–3 hour period focused around the normative times for lunch, before the heated food deteriorates or cools below the optimum temperature. Despite these time challenges and routinisation of work for delivery staff, social interactions between MoW clients and staff were a central feature of the service. On the observed round, participants appeared happy to see the delivery driver and shared brief conversations. Delivery drivers also valued these social opportunities and were clear that this was a key feature of their work. The following section will explore how these social interactions are performed by staff, and how they are interpreted and given meaning by both staff and MoW recipients. The exchanges are necessarily brief encounters, for some this is a brief exchange at the door as food is delivered, but for others who need a little more help, for example, with serving the food, making a hot drink etc, there is time for a slightly longer exchange. All participants described the relationship staff build with them as important and extends their social networks. Relationships are built up over time through ongoing, repeated brief encounters and observations. In these encounters, small snippets of lives are shared, and observations made by staff and participants alike convey reciprocal caring. Participants feel valued and cared for and small kindnesses are noted, building to meaningful relationships. Ann elaborates:

…they don't stand and chat but… they'll say to me ‘we won't be here tomorrow or next week because we're on, I'm on holiday’… just a few words. I mean one day my bell wouldn't ring, and the person that delivered it, he said ‘well I'll do a note for you’ and he wrote out ‘bell not working, please knock’ …and when I spoke to the person on the phone at the Meals on Wheels … I said how helpful he was the person that delivered. The next time I saw him he said ‘oh I got a gold star for doing that’ (Ann)

For staff working within the MoW organisation, relational‐based care was described as an essential part of the service. Ensuring clients felt valued was thought to impact on social connectedness and loneliness, but also encouraged nutritional well‐being through motivating people to eat.

I think tackling loneliness is one of the first ways to support health and wellbeing, especially around nutrition and a lot of these individuals who are feeling lonely won't cook themselves something. They don't have the motivation … and even that five minutes, ten minutes of interaction, plate the meal up for them … the motivation to eat is there because the meal is there so they do tend to eat it… The food is provided but also we're tackling that loneliness, which is such a big challenge for elderly people. (Focus Group participant)

Knowing MoW clients means that if something changes, or if someone is unwell, issues are noted early and referrals made to other agencies. Knowing someone will be visiting every single day is a great reassurance for clients and their families:

So that sort of really kind of emotional, very personal aspect is something that we are able to support with, because if we see things we report them. We're the eyes and the ears. We can help professionals and family members care for their loved ones and really support people in the way that they need to be. (Interview with Manager #2)

### 3.4 Supporting coping capacity during a pandemic

As the pandemic began, all participants described the relief and reassurance they felt to be receiving support from the MoW service during the first lockdown restrictions. They were aware of the challenges other people were facing in maintaining their food security, and the difficulties associated with food shopping as others in society were panic buying food. They were pleased that the service had adapted to keep them safe, for example, by delivering food via a window rather than entering their home and staff wearing masks to protect them from infection. One participant had increased the number of days she now had MoW and had begun to have the additional ‘tea’ service. She had to ‘shield’ for health reasons and was unable to purchase food for herself as she typically did. Reassurance that participants would continue to receive their meals was based on prior experience of reliability as their meals had arrived during periods of bad weather.

Carol described the new protective measures that MoW staff had put in place to protect her including wearing masks and handing food over at the door. She was pleased that she continued to see people every day and receive her hot meal. Others wondered how they would have managed to access food if they did not have the MoW service as during the lockdown they felt that other options would have been limited due to the infection control measures in place. Elsie commented:

Oh, don't ask me. I mean, I haven't thought about it. I don't know. I mean, it would have been all right before you were restricted because I could go out bought stuff or been taken out and buy stuff and my friend would be bringing it in. (Elsie)

Staff were also concerned about the impact the lockdown was having on clients, many of whom were shielding due to their clinical vulnerability. They explained how clients' contacts with the outside world had become very restricted with the MoW delivery being the only contact some older people had. Home assessments by the nutrition team had also moved to a telephone service.

[Clients] have said that it's the only person they see all day, it's the only contact they have with the outer world and a lot of them are scared and knowing that someone's gonna come every day and bring them a meal that's hot and check on them and they're going to have food, has really helped. (Focus group participant)

The following manager indicated the vital role MoW services played to support vulnerable older people, with demand for services escalating, and a sense that they were well placed to step up, compared with areas where there was no similar existing service.

… we can see anecdotally that areas where Meals on Wheels services exist have found their food response to coronavirus easier to support some of those vulnerable in the community.

A lot of the government's funded meals are for a shielded cohort and you have to be able to cook it and there's a cohort of people that cannot cook… we saw unprecedented demand … the number of meals we're delivering to individuals has increased dramatically. (Interview with Manager #3)

## 4 DISCUSSION

The data clearly show that the MoW service was a community asset, having a positive impact in supporting food practices and food security. The service, and in particular, the frontline staff delivering it enabled participants not only to exist, but to flourish, partly through releasing their capacity to enjoy activities such as shopping trips and meeting friends. Thus, vulnerability is not a permanent or fixed state that older people passively move into. Rather vulnerability represents a fluid boundary that people live with, move towards and actively use their agency to seek ways (sometimes with the support of others) to move away from. The beginning of the Covid‐19 pandemic coincided with the data collection of the study, however, continuing the study gave us a unique opportunity to find out how an MoW service supported the food security of older service users during a major societal disruption.

All participants described how they had turned to the MoW service when they struggled with undertaking food practices particularly related to food acquisition and preparation of meals. Arranging to have MoW was perceived by participants to be an agentic act of taking care of themselves that enabled them to continue to access and eat ‘proper healthy meals’ and remain in their own homes (Bonagurio et al., 2022). Modest food stores in the home echoed these changed household food practices as participants were no longer preparing cooked meals for themselves. The limited household food stores potentially reduces household resilience to a major or prolonged service disruption. However, MoW recipients had confidence that service provision would not be affected even when they saw food supply issues resulting from the Covid‐19 pandemic on television. Participants were confident that their food would continue to arrive. For some, this was based on previous experience of services continuing despite adverse weather conditions. The pandemic had presented challenges for the MoW provider in particular with regard to keeping clients safe, while continuing to meet their needs. Participants indicated that they valued the service and appreciated efforts to maintain service delivery.

The relational aspects of the MoW service were clearly important and valued by participants and staff. Relationships were forged through repeated ‘brief encounters’ which enabled participants and those delivering the food to establish reciprocal relationships and ensured older people felt cared for and valued as well as enabling observation of changes to well‐being. This aspect of the service provided social connection and reduced feelings of isolation and loneliness. During the lockdown when older people's social networks were further restricted, having daily visits increased in importance. Older adults are more likely to experience changes to social networks as a result of bereavement and other life changes resulting in negative health and well‐being (Victor & Bowling, 2012) and moving towards food insecurity (Burris et al., 2021). A randomised controlled trial in the US compared those receiving daily MoW with those having weekly deliveries and found daily deliveries reduced feelings of loneliness (Thomas et al., 2015). A recent US study found daily MoW visits impacted loneliness through four mechanisms, a daily safety check (which reassured people that help would be coming if anything went wrong), creating opportunities for social contact, increasing recipients' ability to remain independent and reducing physical risk in those who had mobility issues (Bonagurio et al., 2022). There are no studies of the impact of MoW on loneliness in the UK or on recipient experiences of the non‐nutritional benefits of MoW, but our study may offer some indicators of how loneliness is impacted by MoW, that is through relational care. If MoW reduces feelings of loneliness, then this could have economic benefits by reducing health and social care use, as studies have associated loneliness with increase service use (Wang et al., 2019).

Figure 1 illustrates how an MoW service supports the coping capacity of older people and elaborates on one of the four domains, coping capacity, of the vulnerability model developed by Dickinson et al. (2021). As people become frail, both major and more everyday threats such as difficulties with mobility can accumulate to challenge their food and wider social practices by making it difficult to shop for or cook food. Without external intervention to support food practices, recipients would move towards and potentially into food insecurity. As MoW services are no longer universally available in the UK, further research to explore how older people experiencing challenges to their food practices cope where this service is lacking is required.

One of the strengths of the study is the multimethod design. The collection of visual data supported data from informal interviews to add depth to our understanding of food practices. Adaptation of the design to the pandemic restrictions mean that we were able to capture how older people responded to the crisis as it was unfolding. However, data collection via telephone meant that we were unable to gather the rich data and insights gathered when spending time in someone's home and was a limitation of the data collected during the lockdown period. The study involves a small sample recruited from one MoW provider, further studies could include a range of MoW providers as well as exploring how older people meet their food needs where there is no MoW service.

## 5 CONCLUSION

This study demonstrates the value MoW organisations offer to older people in terms of supporting an individual's coping capacity. With their responsibilities for public health, local authorities considering decommissioning meals on wheels services need to consider the wider benefits a MoW service offers. This is the first study in the UK that has explored older people's perspectives of MoW and shows the positive public health and preventative aspects relating to food security and the advantages of such services in times of individual and societal crisis.

## AUTHOR CONTRIBUTIONS

A Dickinson and W Wills contributed to the design and planning of the study. A Dickinson undertook data collection supported by A Knight and K Geddes, A Dickinson coded the data. Both authors contributed to data analysis and interpretation, drafted the paper, revised the paper in response to reviewer feedback and approved the final manuscript.

## CONFLICT OF INTEREST

The authors declare that they have no conflicts of interest.

## Data Availability

The data that support the findings of this study are available from the corresponding author upon reasonable request.
